# Panoramic lens designed with transformation optics

**DOI:** 10.1038/srep40083

**Published:** 2017-01-06

**Authors:** Huaping Wang, Yangyang Deng, Bin Zheng, Rujiang Li, Yuyu Jiang, Shahram Dehdashti, Zhiwei Xu, Hongsheng Chen

**Affiliations:** 1 Institute of Marine Electronics Engineering, Zhejiang University, Hangzhou 310058, China; 2 State Key Laboratory for Modern Optical Instrumentation, Zhejiang University, Hangzhou 310027, China

## Abstract

The panoramic lens is a special kind of lens, which is applied to observe full view. In this letter, we theoretically present a panoramic lens (PL) using transformation optics method. The lens is designed with inhomogeneous and anisotropic constitutive parameters, which has the ability to gather light from all directions and confine light within the visual angle of observer. Simulation results validate our theoretical design.

Since the human visual angle is about 120°, it is impossible for a person to get a full view of the environment. Usually, people use panoramic annular lens^1^,^2^,^3^,^4^, multi-camera system^5^,^6^ or stitching techniques^7^,^8^ to achieve panorama. Although all these methods can get a panoramic view, they still have some defects. For example, due to the limit of average visual angle, the observer has to move his/her head in order to get a full view if an annular lens is used directly. Otherwise, sophisticated electronic devices and complicated algorithms are required for the latter two methods, and the observation is affected by the limitation of energy and reliability of electronic devices. In this letter, we present a novel way to design panoramic lens (PL), which may has the ability to make up above shortcomings.

Transformation optics (TO)^9^,^10^,^11^,^12^,^13^,^14^ is a method that can control propagation of light due to the invariance of Maxwell equation under geometrical transformation^15^. Since it is first proposed, TO has found wide applications in designing novel electromagnetic (EM) devices, including invisibility cloak^16^,^17^,^18^,^19^, hyper-lens^20^,^21^, waveguide^22^,^23^,^24^, superscatters^25^,^26^,^27^, etc. In this peper, we theoretically design a novel lens, which can not only take panoramic photos, but also compress the image, for it can fit the visual angle of the average person. Light in all directions will be confined within the visual angle of observer who equipped this lens. Hence, comparing to ordinary lens, this panoramic lens based on transformation optics has some unique advantages. Simulations are conducted to verify the performance.

As shown in Fig. 1, the structure of PL is a transparent hollow cylinder, where the green regions ① and ③ are filled with air, the blue region ② represents the lens, four white arrows denote light rays coming from different directions, the visual angle is *θ*_0_ which equals to 120° for average person, and the black thick line denotes the screen in front of the observer. Without the PL, all the light rays will propagate straightly. Since light rays II and III are incident from the backside, they cannot come into view of the observer. Whereas, if PL is adopted, all the light rays will change their trajectories and hit the screen with incident angles changing from −*θ*_0_/2 to *θ*_0_/2. Therefore, with the help of PL, the observer can see a panoramic view without moving his/her head. It should be noted that after the guidance of PL, the light rays coming from all directions are confined into the visual angle without passing through region ③. For this reason, observer himself/herself in region ① doesn’t affect the imaging quality.

## Methods

Based on the light trajectory inside the PL, we use transformation optics to satisfy the above requirement. (*r, θ, z*) and (*r*′, *θ*′, *z*′) are the coordinates in virtual space and physical space, respectively. In cylindrical coordinate system, coordinate transformation shown in Fig. 2 is governed by the following transformation function


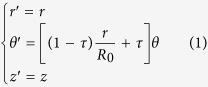


where the compression ratio *τ* ∈ [0, 1], *R*_0_ is the radius of PL, and *θ* ∈ (−*π, π*).

**Figure 1:**
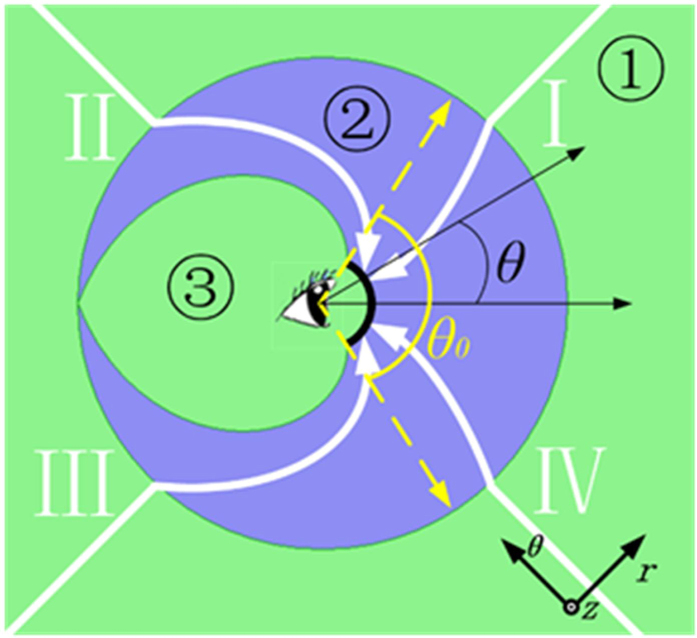
Schematic illustration of the PL, where the green regions ① and ③ are filled with air, the blue region ② represents the lens, four white arrows denote light rays coming from different directions, *θ*_0_ is the visual angle of average person, and the black thick line denotes the screen in front of the observer.

**Figure 2:**
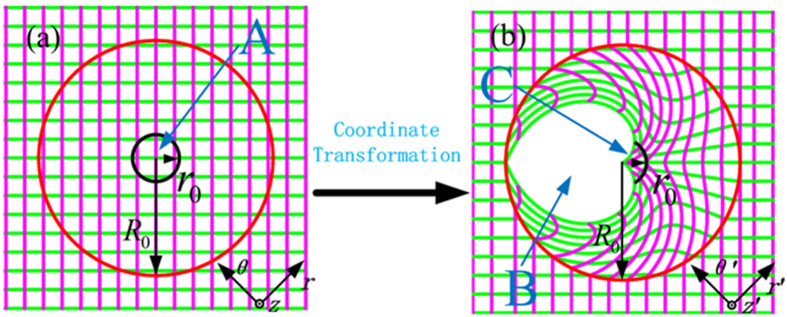
Illustration of the coordinate transformation, where the area surrounded by red circle in virtual space (**a**) is transformed into physical space (**b**). *r*_0_ is radius of screen, *R*_0_ is radius of PL. A, B and C represent different regions.

In Fig. 2(a), the black circle denotes a screen with radius *r*_0_. In this virtual space, the observer in region A needs to rotate his/her head in order to get a full view because of the annular shape of the screen. After the coordinate transformation, the screen is changed into an arc in physical space, where the corresponding angle is chosen as the visual angle of average person. Besides, after transformation, a blank region B is created, while the circular region A is transformed into the sector region C. If regions B and C are combined, we can get the whole hollow region ③ in the cylindrical PL, as shown in Fig. 1.

**Figure 3:**
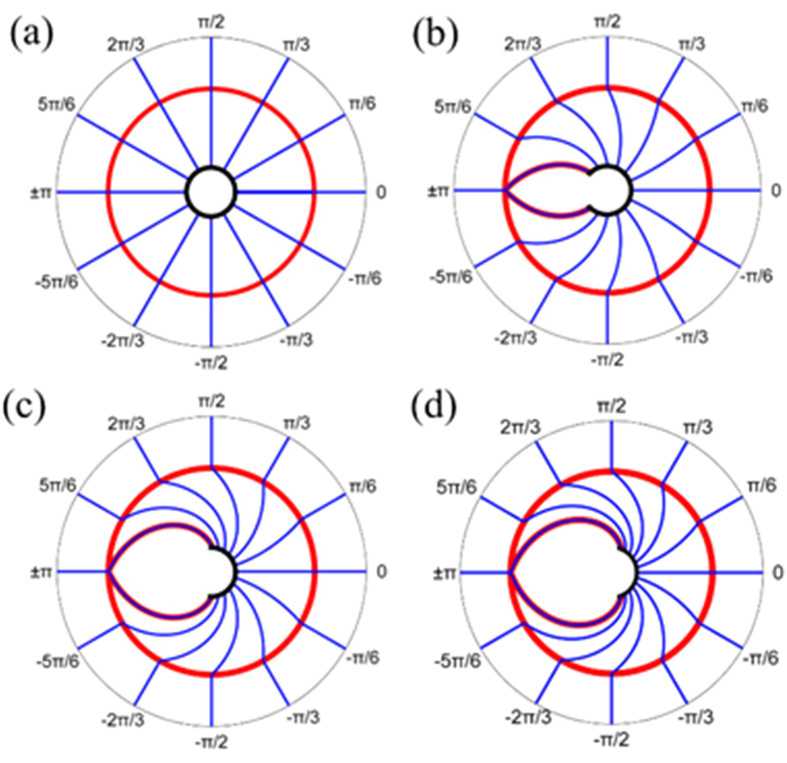
The propagation of light rays for *τ* = 1 (**a**), 2/3 (**b**), 1/3 (**c**), and 1/6 (**d**), respectively. Red circles: the interface between PL and air. Black circle/arcs: screen for observer.

Figure 3 shows the propagation of light rays from different directions for different compression ratios with *R*_0_ = 1(m) and *r*_0_ = 0.2(m), where the red circles denote the edge of PL, and the black circle/arcs around the center represent the screen. In Fig. 3(b–d), regions B and C from Fig. 2(b) are combined, so the screen is also a part of boundary to separate the hollow region and PL. The compression is more pronounced for a smaller *τ*. Specially, if *τ* equals to 1, there is no difference between virtual and physical spaces, hence the propagation of the light rays is not changed, as shown in Fig. 3(a). Because the visual angle *θ*_0_ for average person is 120°, the compression ratio *τ* is chosen as 1/6 in the following discussion. Thus the light rays from all directions can be guided and confined within the visual angle, and a full view can be obtained.

The relative permittivity 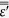 and relative permeability 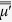 of PL can be achieved with transformation optics method:


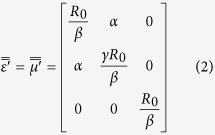


where 
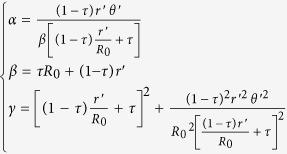
.

**Figure 4:**
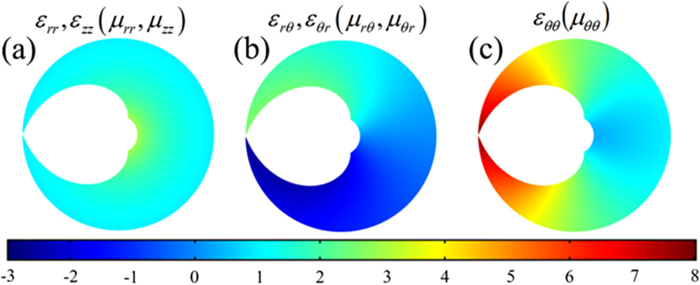
Permittivity and permeability distributions of PL with *R*_0_ = 1(m), *r*_0_ = 0.2(m), and *τ* = 1/6.

From Eq. (2), 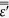 and 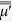 are equal, and they are both dependent on *r*′ and *θ*′. And their distributions are shown in Fig. 4 with *R*_0_ = 1(m), *r*_0_ = 0.2(m) and *τ* = 1/6.

## Results

**Figure 5:**
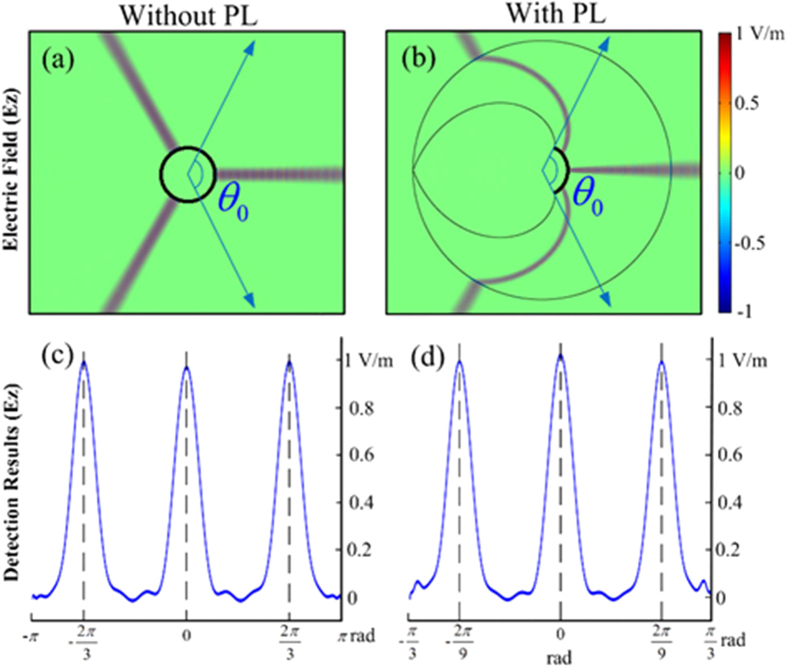
Electric field distributions without (**a**) and with (**b**) PL, respectively. Three Gaussian beams at 20 GHz are incident from different directions. (**c**,**d**) Electric field distributions at the screen.

Based on the above results, we use Comsol Multiphysics^®^ to verify the performance of PL. Three Gaussian beams at 20 GHz are incident from different directions and the areas surrounded by screens are filled with absorbing materials. Figure 5(a) and (b) show the electric field distributions without and with PL, respectively. Clearly, without PL, two beams coming from backside cannot come into the observer’s eyes. But if PL is equipped, the two beams are compressed, and they propagate in curved trajectories, and hit the screen. Thus the observer can get a panoramic view without moving his/her head. Besides, due to the relation *θ*′ = *θ*/3, the incident angles of three Gauss beams are compressed from (0, 2*π*/3, −2*π*/3) to (0, 2*π*/9, −2*π*/9), as shown by the corresponding electric field distributions at the screen in Fig. (c,d). Hence, the simulation results validate our theoretical design of PL.

**Figure 6:**
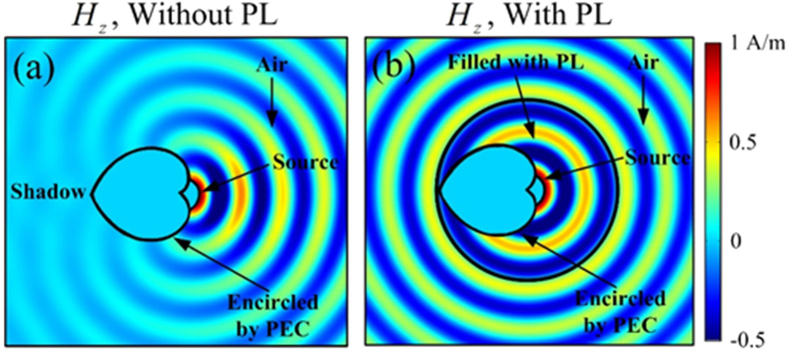
*Hz* distributions when a source is placed on the screen without (**a**) and with (**b**) PL. The hollow regions are encircled by PEC.

Utilizing the reversibility of light, simulations are carried out to test the overall performance, where a TM polarized source is placed on the screen. Since perfect electric conductor (PEC) is used to encircle the hollow region B in Fig. 2(b), this will partly prevent EM wave propagating to the left, as shown in Fig. 6(a). But with the help of PL, as shown in Fig. 6(b), EM wave can bypass the hollow region and propagate radially, and the original shadow in Fig. 6(b) disappears. Thus, PL has a great effect on EM wave distribution in the surrounding air. Since a cylindrical wave is excited by the source in Fig. 6(b), the proposed PL works for light coming from all directions.

## Discussion

The previous results show the performance of our designed panoramic lens, which has the ability to gather the light from all direction and to confine them into the front of the observer within the visual angle. However, it requires the permittivity and permeability to be both anisotropic and inhomogeneous simultaneously. In the following, we discuss the possibility of experimental realization of this kind of lens. From Eq. (2), we can see the constant tensors have coupling component beside the diagonal. It can be expressed as diagonal ones by rotating the original coordinate system with a certain angle *φ* into a new local principal coordinate system similar to that in ref. 28. Since the constitutive parameters shown by Eq. (2) are spatially inhomogeneous, the rotation angles will also vary with different position. Consider the TM case (the magnetic field along the z direction) as example, we need to consider the permeability along the z direction and the permittivity along the other two directions. Furthermore, we can simplify the parameter into a non-magnetic form by setting the permeability to be unity while keeping the refractive index unchanged. We can finally obtain the required permittivity and permeability in their local principle coordinate system to be:


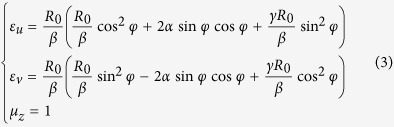


where 
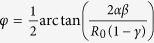
. We can see from Eq. (3) that the complexity of the parameters is much reduced. It should be noted that since the parameters are set to be non-magnetic, there will be impedance mismatch at the boundary of our designed lens. It will cause some reflection at the boundary of the lens but the refracted lights in the lens will still keep the same trajectory as designed.

**Figure 7:**
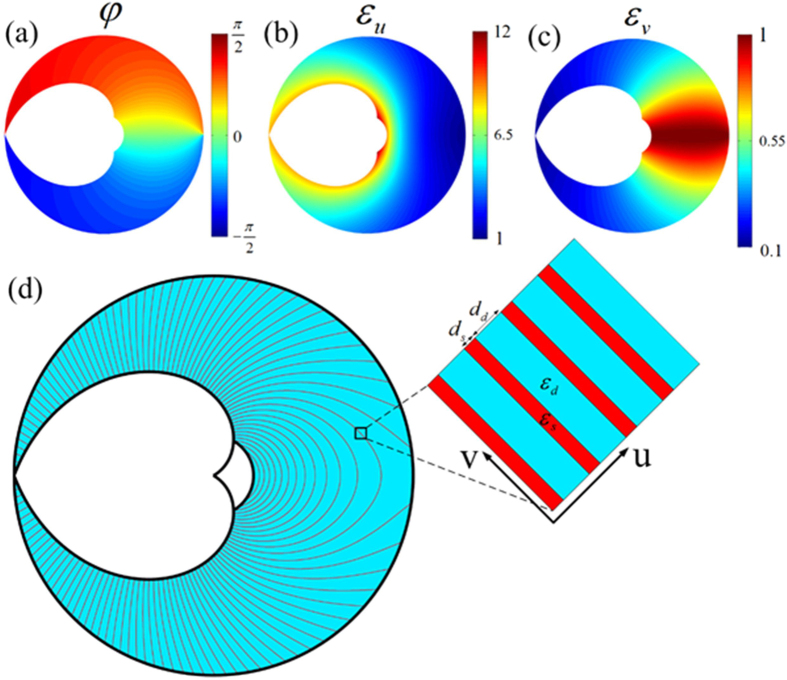
(**a–c**) The spatial distribution of ***φ**, **ε***_***u***_ and ***ε***_***v***_. (**d**) The schematic of layered system to achieve the required parameters.

In Fig. 7 (a–c), we plot the distribution of the rotation angles *φ*, permittivity *ε*_*u*_ and *ε*_*v*_ for the simplified parameters, respectively. It shows significant anisotropy for the permittivity. As shown by ref. 29, a thin layered system with homogeneous and isotropic permittivity can be treated as a single anisotropic medium. Making use of this concept, the proposed lens has the possibility to be practically realized. Figure 7(d) shows the schematic of the layered structure used for the designed lens. When the layers are sufficiently thin, the permittivity of the structure can be treated as:


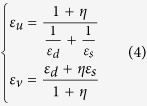


where *η* = *d*_*s*_/*d*_*d*_ is the ratio of the two layers’ widths. For instance, if we consider a silver/dielectric layered structure and suppose an infrared frequency with *ε*_*s*_ = −1000 for silver. Since the absolute value of the permittivity *ε*_*s*_ is very large, the ratio of *η* need to be extremely small to satisfy Eq. (4). Thus, we can get the approximated solution of Eq. (4) to be *ε*_*d*_ ≈ *ε*_*u*_ as well as *η* ≈ (*ε*_*u*_ − *ε*_*v*_)/(*ε*_*v*_ − *ε*_*s*_). The inhomogeneous permittivity of the dielectric can be obtained by drilling holes in dielectric substrate^30^ while the thickness of the silver layer can be controlled to achieve the different ratios. With this mentioned layered system, this proposed panoramic lens has the potential to be realized.

## Conclusion

In conclusion, we theoretically design a panoramic lens based on transformation optics, which can guide light from all directions to the screen set in front of observer. Simulations are carried out to validate its performance. Because PL can provide a great convenience for the user and works without battery or sophisticated electronic equipment, it is promising in wide applications, like photography, electronic maps, detection, etc.

## Additional Information

**How to cite this article**: Wang, H. *et al*. Panoramic lens designed with transformation optics. *Sci. Rep.* **7**, 40083; doi: 10.1038/srep40083 (2017).

**Publisher's note:** Springer Nature remains neutral with regard to jurisdictional claims in published maps and institutional affiliations.
